# Zusammenhang von Mundgesundheitskompetenz und -verhalten mit physischer Mundgesundheit. Welche Rolle kann die zahnmedizinische Ausbildung spielen?

**DOI:** 10.1007/s00103-023-03793-2

**Published:** 2023-11-10

**Authors:** Daniel R. Reißmann, Ragna Lamprecht, Uwe Koch-Gromus, Katrin Borof, Christopher Kofahl, Martin Härter, Julie Büschel, Volker Harth, Hanno Hoven, Bärbel Kahl-Nieke, Thomas Beikler, Guido Heydecke, Ghazal Aarabi

**Affiliations:** 1 Poliklinik für Zahnärztliche Prothetik, Zentrum für Zahn‑, Mund- und Kieferheilkunde, Universitätsklinikum Hamburg-Eppendorf, Hamburg, Deutschland; 2 Klinik für Zahnärztliche Prothetik, Department für Zahn‑, Mund- und Kieferheilkunde, Universitätsklinikum Freiburg, Freiburg, Deutschland; 3 Poliklinik für Zahnärztliche Prothetik und Werkstoffkunde, Universitätsklinikum Leipzig, Leipzig, Deutschland; 4 Institut und Poliklinik für Medizinische Psychologie, Zentrum für Psychosoziale Medizin, Universitätsklinikum Hamburg-Eppendorf, Hamburg, Deutschland; 5 Poliklinik für Parodontologie, Präventive Zahnmedizin und Zahnerhaltung, Zentrum für Zahn‑, Mund- und Kieferheilkunde, Universitätsklinikum Hamburg-Eppendorf, Martinistr. 52, 20246 Hamburg, Deutschland; 6 Institut für Medizinische Soziologie, Zentrum für Psychosoziale Medizin, Universitätsklinikum Hamburg-Eppendorf, Hamburg, Deutschland; 7 Zentralinstitut für Arbeitsmedizin und Maritime Medizin, Universitätsklinikum Hamburg-Eppendorf, Hamburg, Deutschland; 8 Poliklinik für Kieferorthopädie, Zentrum für Zahn‑, Mund- und Kieferheilkunde, Universitätsklinikum Hamburg-Eppendorf, Hamburg, Deutschland

**Keywords:** Mundgesundheitskompetenz, Mundgesundheitsverhalten, Mundgesundheit, Epidemiologie, Risikofaktoren, Oral health literacy, Oral health behaviour, Oral health, Epidemiology, Risk factors

## Abstract

**Hintergrund:**

Mundgesundheit ist ein wesentlicher Bestandteil der allgemeinen Gesundheit und des Wohlbefindens. Sie wird von vielen Faktoren beeinflusst. Dazu zählen insbesondere individuelle Aspekte wie Mundgesundheitskompetenz und -verhalten. Ziel der Studie war die Bestimmung des konkreten Zusammenhangs zwischen Mundgesundheitskompetenz und -verhalten mit physischer Mundgesundheit.

**Methoden:**

In dieser bevölkerungsbasierten Querschnittsstudie wurden Daten von insgesamt 5510 Personen, welche von 2016 bis 2018 in die Hamburg City Health Study (HCHS) eingeschlossen wurden, mit einem durchschnittlichen Alter von 62,1 Jahren und einem Frauenanteil von 50,7 % ausgewertet. Die physische Mundgesundheit wurde mit den 14 Items des Physical Oral Health Index (PhOX) erhoben. Zur Bestimmung von Mundgesundheitskompetenz und -verhalten wurde ein neu entwickelter Fragebogen mit 10 Aspekten basierend auf dem Oral Health Literacy Adult Questionnaire und der 5. Deutschen Mundgesundheitsstudie genutzt.

**Ergebnisse:**

Der Summenwert der 10 Fragen in Bezug auf Mundgesundheitskompetenz und -verhalten korrelierte signifikant mit dem PhOX-Summenwert (r = 0,23; *p* < 0,001). Ein Anstieg von einem Punkt des Gesamtwerts zu Mundgesundheitskompetenz und -verhalten war assoziiert mit einer Erhöhung des PhOX-Summenwerts um durchschnittlich 1,45 Punkte. Dies reduzierte sich nur unwesentlich nach Einbeziehung von potenziellen Confoundern wie Alter und Bildung.

**Schlussfolgerung:**

Höhere Mundgesundheitskompetenz und besseres entsprechendes Verhalten sind assoziiert mit einer besseren Mundgesundheit. Damit bilden Mundgesundheitskompetenz und -verhalten wichtige Zielgrößen in der zahnmedizinischen Ausbildung zur effizienten und nachhaltigen Verbesserung der Mundgesundheit in der Allgemeinbevölkerung.

## Abstract (en)

**Background:**

Oral health is an essential component of a person’s general health and well-being. It is influenced by many factors. These include individual aspects such as oral health literacy and oral health behaviour. The aim of this study was to investigate the association between oral health literacy and behaviour with physical oral health.

**Methods:**

In this population-based cross-sectional study, data of 5510 subjects enrolled in the Hamburg City Health Study (HCHS) from 2016 to 2018 with a mean age of 62.1 years and 50.7% women were evaluated. Physical oral health was assessed using the 14-item Physical Oral Health Index (PhOX). A newly developed 10-item questionnaire based on the Oral Health Literacy Adult Questionnaire and the 5th German Oral Health Study were used to determine oral health literacy and behaviour.

**Results:**

The sum score of the 10 questions related to oral health literacy and behaviour significantly correlated with the PhOX sum score (r = 0.23; *p* < 0.001). An increase of one point in the total score of oral health literacy and behaviour was associated with an increase in the PhOX sum score of 1.45 points on average. This association decreased only marginally after integrating potential confounders such as age and education.

**Conclusion:**

Higher oral health literacy and better oral health behaviour are associated with better physical oral health. Oral health literacy and behaviour should be important targets in dental education to efficiently and sustainably improve the oral health of the general population.

## Einleitung

Mundgesundheit ist ein wesentlicher Bestandteil der allgemeinen Gesundheit und des Wohlbefindens. Sie kann unterteilt werden in die physische und die psychosoziale Mundgesundheit. Optimale Mundgesundheit wird nicht nur als Abwesenheit von Krankheiten beschrieben, sondern auch als die Fähigkeit des Einzelnen, Ernährung sowie Kommunikations- und Sinnesfunktionen ohne Schmerzen ausführen zu können [1]. Im Jahr 2019 waren etwa 3,5 Mrd. Menschen weltweit von oralen Erkrankungen wie Tumoren in der Mundhöhle, Karies oder parodontalen Entzündungen betroffen [2]. Bleiben die zahnbezogenen Erkrankungen unbehandelt, führen sie mit einer hohen Wahrscheinlichkeit zu Zahnverlust und Zahnlosigkeit, was wiederum in einer schlechteren Kaufunktion resultiert und die Lebensqualität verringern kann [3, 4]. Die Weltgesundheitsorganisation (WHO) hat im Jahr 2022 eine „Globale Strategie zur Mundgesundheit“ entwickelt mit dem Ziel, die mundgesundheitliche Ungleichheit zu verringern. Große Bedeutung hat dabei neben einem Public-Health-Ansatz auch die Verlagerung von der kurativen zur präventiven zahnärztlichen Versorgung [5]. Studien wie die 5. Deutsche Mundgesundheitsstudie (DMS V) zeigen beispielsweise, dass die aktuellen Anstrengungen in der Prävention große Erfolge erzielt haben. Diese Maßnahmen betrafen sowohl das Verhalten der Menschen (Verhaltensprävention) als auch die Verhältnisse (Verhältnisprävention) und haben zu einer starken Abnahme der Prävalenz der häufigsten oralen Erkrankungen geführt. Bei jüngeren Erwachsenen (35- bis 44-Jährigen) ist die Anzahl der Zähne mit Karieserfahrung seit 1997 um 30 % zurückgegangen und die Prävalenz der schweren Parodontalerkrankungen hat sich in Deutschland zwischen 2004 und 2014 halbiert. Nur noch jeder achte Senior (65- bis 74-Jährige) war im Jahr 2014 zahnlos [6]. Jedoch reichen diese durchaus bemerkenswerten Erfolge bei Weitem noch nicht aus, die WHO-Ziele zur Verbesserung der Mundgesundheit als erreicht zu betrachten.

Mundgesundheit wird durch zahlreiche Risikofaktoren beeinflusst. Dazu zählen soziodemografische Faktoren (z. B. Alter, Geschlecht, Bildung, Einkommen), genetische Prädisposition, orale Faktoren (z. B. Zusammensetzung des Speichels und Biofilms) und Verhalten (z. B. Ernährung und Mundhygiene; [7]). Wesentlich für die Entstehung von oralen Erkrankungen ist das Mundgesundheitsverhalten. Hierzu zählen bei der Ernährung insbesondere die Frequenz und Menge konsumierter niedermolekularer Kohlenhydrate, speziell Zucker, da diese in Zusammenhang mit der Entstehung von Karies stehen. Darüber hinaus ist regelmäßiges Zähneputzen positiv mit allen Aspekten der Mundgesundheit assoziiert [6]. Voraussetzung für das Mundgesundheitsverhalten ist die Mundgesundheitskompetenz. Dies bezeichnet die Fähigkeit, grundlegende Gesundheitsinformationen und -angebote zu erschließen und zu verstehen, um angemessene Entscheidungen bezüglich der Mundgesundheit zu treffen [8]. Mundgesundheitsverhalten und -kompetenz sind demzufolge essenziell für eine gute Mundgesundheit, wobei nach diesem Verständnis die Gesundheitskompetenz eine wesentliche Voraussetzung für das daraus abgeleitete Verhalten darstellt.

Mehrere gesundheitspsychologische Modelle betonen einen positiven Zusammenhang zwischen Mundgesundheitskompetenz, Mundgesundheitsverhalten und Mundgesundheit [9]. Studien zeigen beispielsweise, dass Patient:innen mit niedriger Mundgesundheitskompetenz im Vergleich zu Patient:innen mit hoher Mundgesundheitskompetenz ein höheres Risiko aufweisen, an Parodontitis zu erkranken (29 % vs. 7 %) und eine niedrigere Anzahl gefüllter Zähne zu haben (3,3 vs. 4,0; [10]). Die DMS V zeigt zudem einen positiven Zusammenhang zwischen Mundgesundheit und Mundgesundheitsverhalten. Während sich die Anzahl der 65- bis 74-Jährigen mit einer guten Mundgesundheit im Vergleich zum Jahr 1997 fast verdreifacht (32 %) hat, legten auch wesentlich mehr ein adäquates Mundgesundheitsverhalten an den Tag; zumindest bezüglich der regelmäßigen Inanspruchnahme von zahnärztlichen Kontrollen (90 %) und professioneller Zahnreinigung (40 %; [6]). Jedoch ist die derzeitige Studienlage zu diesem Thema unzureichend und die bestehenden Studien zeigen zudem insgesamt heterogene Ergebnisse [11, 12].

Zur Verbesserung von Mundgesundheitskompetenz und -verhalten sowie infolge der Mundgesundheit selbst existieren verschiedene Ansätze [13]. Wichtige allgemeine Bausteine sind die Gewährleistung eines ungehinderten Zugangs zu zahnmedizinischen Leistungen, das Angebot von leicht verständlichem, gegebenenfalls mehrsprachigem Informationsmaterial und einer kostenfreien Patientenberatung durch die Vertragszahnärzteschaft. Eine zentrale Bedeutung hat die Kommunikation zwischen Patient:innen und Zahnärzt:innen sowie anderem Fachpersonal wie zahnmedizinischen Prophylaxeassistent:innen oder Dentalhygieniker:innen [14]. Dabei kommt den Zahnärzt:innen sicherlich eine besondere Rolle zu, da sie in der Regel alle Patienten sehen und nicht nur die, welche sich schon für präventive Maßnahmen wie Zahnreinigung und Mundhygieneinstruktionen entschieden haben.

Eine ausführliche und verständliche Aufklärung unter Einbezug der psychischen und sozialen Situation der Patient:innen bildet die Basis für ein gutes Vertrauensverhältnis sowie die notwendige Adhärenz, um Mundgesundheitskompetenz und -verhalten dauerhaft positiv zu beeinflussen. Grundlage dafür ist eine soziale Kompetenz seitens der Zahnärzt:innen und eine adäquate Ausbildung bereits im Zahnmedizinstudium. Daher sollten die Themen „Mundgesundheitskompetenz“ und „Mundgesundheitsverhalten“ sowie die Frage, wie Patient:innen hierüber informiert und zu Verhaltensänderungen motiviert werden können, einen wesentlichen Bestandteil im Zahnmedizinstudium darstellen. Dadurch können die Studierenden höhere Kompetenzen entwickeln, dieses Wissen an die Patient:innen weiterzugeben und somit für eine bessere Mundgesundheit in der Bevölkerung zu sorgen. Grundlage für diesen Ansatz ist aber ein eindeutiger Nachweis, dass Mundgesundheitskompetenz und -verhalten und physische Mundgesundheit tatsächlich zusammenhängen. Die Datenlage dazu war bisher nicht ausreichend.

Ziel dieser Studie war es, die Zusammenhänge zwischen physischer Mundgesundheit und Mundgesundheitskompetenz und -verhalten zu untersuchen.

## Methoden

### Studienpopulation, Studiendesign und Setting

Die folgende Querschnittsstudie wurde als Teil der Hamburg City Health Study (HCHS) am Universitätsklinikum Hamburg-Eppendorf (UKE) in Hamburg, Deutschland, durchgeführt [15]. Die HCHS ist eine Kohortenstudie, die auf eine Gesamtstichprobe von insgesamt 45.000 Personen zwischen 45 und 74 Jahren aus der Hamburger Allgemeinbevölkerung zielt. Mittels der HCHS sollen Risikofaktoren und wichtige Prädiktoren der relevantesten Bevölkerungskrankheiten identifiziert werden. Die potenziell Teilnehmenden wurden und werden als Zufallsstichprobe aus den Daten des Einwohnermeldeamts ausgewählt, angeschrieben und um Mitwirkung gebeten. Eingeschlossen werden Personen mit ausreichenden Deutschkenntnissen und physischer wie psychischer Eignung zur Teilnahme.

Für die vorliegende Auswertung wurden die Daten der Baseline-Untersuchung der ersten 10.000 Teilnehmenden der HCHS genutzt, welche zwischen Februar 2016 und November 2018 rekrutiert wurden. Diese Stichprobengröße erlaubt auch in alters- und geschlechtsspezifischen Subgruppen (ca. *n* = 1000) die Identifikation von Korrelationen im Bereich von r = 0,10 („kleiner“ Effekt nach Cohen [16]) mit einer Power von 90 % bei einem Typ-I-Fehler von 0,05. Basierend auf einer Streuung der Werte für die physische Mundgesundheit in der Zielpopulation (Personen ohne zahnärztlichen Behandlungsbedarf) von SD = 10 [17] und der Definition eines klinisch relevanten Unterschieds bei einer Effektgröße von d = 0,50 [18] („mittlerer“ Effekt nach Cohen [16]) kann ein Unterschied von 5 Punkten bezüglich der Mundgesundheitskompetenz und des -verhaltens mit einer Power von 90 % bei einem Typ-I-Fehler von 0,05 in Subgruppen von *n* = 265 nachgewiesen werden. Bei insgesamt 5510 Personen konnte eine komplette zahnärztliche Untersuchung durchgeführt werden.

### Erfassung der physischen Mundgesundheit

Die physische Mundgesundheit wurde in einer zahnärztlichen Untersuchung von geschultem und zertifiziertem Studienpersonal mittels des Physical Oral Health Index (PhOX; [17]) erhoben. Der PhOX besteht aus 14 Items, die alle relevanten Strukturen und Zustände der Mundgesundheit anhand von 5 Subskalen abdecken (*Zähne/Stützzonen, intraorales Weichgewebe, extraorales Weichgewebe und Kiefer, Funktion, Wahrnehmung*). Für jedes Item wurden die spezifische Struktur und der Zustand auf einer 5‑stufigen ordinalen Bewertungsskala von 0 bis 4 auf der Grundlage vordefinierter Kriterien bewertet. Die PhOX-Summen- und Subskalenwerte wurden als Summe der entsprechend gewichteten Items mit Gewichtungsfaktoren von 1 bis 3 berechnet. Dementsprechend konnten die PhOX-Summenwerte zwischen 0 und 100 Punkten liegen, wobei niedrigere Werte für eine schlechtere physische Mundgesundheit und 100 Punkte für die bestmögliche physische Mundgesundheit stehen. Die Wertebereiche für die Summenwerte der 5 Subskalen unterscheiden sich aufgrund der Anzahl der Items und der jeweiligen Gewichtungsfaktoren (*Zähne/Stützzonen*: 0–44, *intraorales Weichgewebe*: 0–16*, extraorales Weichgewebe und Kiefer*: 0–12*, Funktion*: 0–16*, Wahrnehmung*: 0–12). Zusätzlich wurde aus der Anzahl der kariösen (D), fehlenden (M) und gefüllten oder überkronten (F) Zähne über den Summenwert der DMF‑T [19] berechnet.

### Erfassung von Mundgesundheitskompetenz und -verhalten

Mundgesundheitskompetenz und -verhalten wurden papierbasiert mittels Fragebogen erfasst. Die Fragen zur Mundgesundheitskompetenz stammen aus dem validierten Oral Health Literacy Adult Questionnaire (OHL-AQ; [20]). Die ausgewählten Fragen sollten dabei alle Bereiche der Mundgesundheitskompetenz abdecken, also Wissen, Entscheidung und Anwendung. Die Fragen zum Mundgesundheitsverhalten wurden aus der DMS V [6] ausgewählt. Dies führte zu insgesamt 17 Fragen. Von diesen waren 7 Fragen primär deskriptiv (z. B. ob schon einmal eine Parodontitistherapie durchgeführt wurde, ob eine professionelle Zahnreinigung regelmäßig in Anspruch genommen wurde oder ob ein Bonusheft vorliegt) und wurden nur zur Beschreibung der Studienpopulation genutzt. Bei 10 Fragen konnten die Antworten bezüglich Mundgesundheitskompetenz und -verhalten eindeutig dichotomisiert werden (positiv/negativ). Im Folgenden werden diese 10 Fragen als Oral Health Literacy (OHL) zusammengefasst.

Aus den dichotomisierten Antworten für OHL wurde ein Summenwert gebildet, welcher von 0 bis 10 reichen konnte, wobei höhere Werte für bessere Mundgesundheitskompetenz und -verhalten stehen. Bei bis zu 2 fehlenden Werten wurden diese durch den intraindividuellen Median ersetzt, da davon auszugehen ist, dass das zu messende Konstrukt ausreichend eindimensional ist. Bei mehr als 2 fehlenden Werten wurde kein Summenwert gebildet. Zusätzlich wurden Subskalen für *Verhalten* (3 Fragen) und *Kompetenz* (7 Fragen) gebildet.

Die Anzahl fehlender Werte war insgesamt gering. Alle 10 Fragen wurden von 3965 Teilnehmenden (72,0 %) vollständig beantwortet. Bei 1132 Teilnehmenden (20,5 %) fehlten die Informationen für eine oder 2 Fragen. Diese konnten imputiert werden. Somit konnten bei 92,5 % der Teilnehmenden die Summenwerte berechnet werden. Bei 413 Teilnehmenden (7,5 %) lagen mit mindestens 3 fehlenden Werten nicht ausreichend Informationen zu den 10 Fragen vor, weshalb diese aus den Analysen für OHL ausgeschlossen werden mussten.

### Erfassung von soziodemografischem, sozioökonomischem und psychosozialem Status

Alle Teilnehmenden erhielten vor dem Besuch im Studienzentrum einen Fragebogen zur Selbstauskunft, der Informationen zu soziodemografischen Merkmalen wie Alter, Geschlecht und Lebensstil enthielt. Das Bildungsniveau wurde nach den Kriterien des International Standard Classification of Education (ISCED; [21]) erhoben und eingestuft. Für die Erfassung der gesundheitsbezogenen Lebensqualität wurde die deutsche Version des SF‑8 (Short Form‑8 Health Survey) eingesetzt [22]. Aus den 8 Fragen wurde entsprechend den Vorgaben jeweils ein Wert für die *physische Lebensqualität* und für die *psychische Lebensqualität* berechnet. Ein Wert von 50 entspricht dabei dem Bevölkerungsdurchschnitt, höhere Werte stehen für bessere und niedrigere Werte für eine schlechtere Lebensqualität.

### Statistische Analysen

Im ersten Teil der Analysen erfolgte die Beschreibung der Studienpopulation über Mittelwerte mit Standardabweichung (SD) für kontinuierliche Daten (z. B. Alter, DMFT, PhOX, OHL, SF-8) und Proportionen mit Prozentwerten für kategoriale Daten (z. B. Geschlecht, Beschäftigungssituation, Parodontitistherapie) und ordinale Daten (Schulbildung).

Im zweiten Teil wurden die Antworten der einzelnen Fragen bezüglich der PhOX-Summenwerte verglichen. Dazu wurden die dichotomisierten Antworten der Fragen des OHL genutzt, welche entweder als korrekt bewertet wurden oder als nicht korrekt bzw. nicht gewusst. Für beide Antwortoptionen wurden jeweils die Mittelwerte und die Standardabweichungen (SD) der PhOX-Summenwerte berechnet und mittels *t*-Test für unabhängige Stichproben auf statistische Signifikanz verglichen. Für die Abschätzung der Stärke des Unterschieds wurde die standardisierte Effektgröße (Cohens *d*) bestimmt [16]. Bei *d* = 0,2 geht man von einem kleinen, bei *d* = 0,5 von einem moderaten und bei *d* = 0,8 von einem großen Effekt aus.

Im dritten Teil erfolgte die Analyse der Summenwerte für Mundgesundheitskompetenz und -verhalten bzw. der beiden Subskalen *Verhalten *und *Kompetenz*. Dazu kamen Pearson-Produkt-Moment-Korrelationen zum Einsatz. Die Korrelationskoeffizienten wurden mit Richtwerten verglichen, um die Stärke des Zusammenhangs zu bestimmen. Entsprechend stellen Koeffizienten von 0,1 einen kleinen, 0,3 einen mittleren und 0,5 einen großen Effekt oder Zusammenhang dar [16].

Letztlich wurden multivariable lineare Regressionsmodelle gerechnet, um die Zusammenhänge auch für potenzielle Confounder wie Soziodemografie, Bildung und Beschäftigungssituation statistisch kontrollieren zu können.

Die Analysen wurden mit der Statistiksoftware Stata Version 17.0 (StataCorp., College Station, TX, USA) durchgeführt.

## Ergebnisse

### Charakteristik der Teilnehmenden

Die 5510 Teilnehmenden in dieser Studie hatten ein Durchschnittsalter von 62,1 Jahren (SD = 8,4; Tab. 1). Der Anteil der Frauen betrug 50,7 %. Knapp die Hälfte (49,8 %) hatte eine mindestens 12-jährige Schulbildung, nur 2,7 % waren ohne Schulabschluss. Die meisten Teilnehmenden waren erwerbstätig, 38,4 % in Vollzeit und 19,7 % in Teilzeit.

**Table 1:** 

Charakteristika	Ausprägung/Umfang
	Mittelwert (SD) oder *n* (%)
*Demografie*	
Alter	62,1 (8,4)
Geschlecht (weiblich)^a^	2682 (50,7)
*Schulbildung*^b^	
Mind. 12 Jahre Schule	2551 (49,8)
10 Jahre Schule	1421 (27,7)
8 Jahre Schule	1010 (19,7)
Ohne Abschluss	140 (2,7)
*Beschäftigungssituation*^c^	
Vollzeitbeschäftigt	1900 (38,4)
Teilzeitbeschäftigt	974 (19,7)
Altersteilzeit oder Ruhestand	74 (1,5)
Arbeitssuchend, in Ausbildung oder Fortbildung	1996 (40,4)
*Lebensqualität*^d^	
SF‑8 physische Lebensqualität	50,3 (8,0)
SF‑8 psychische Lebensqualität	53,6 (7,6)
*Mundgesundheitskompetenz und -verhalten*	
Parodontitistherapie^e^	2197 (43,5)
Professionelle Zahnreinigung^f^	3804 (72,8)
Bonusheft^g^	3451 (66,5)
OHL gesamt^h^	8,2 (1,3)
OHL-Verhalten^h^	2,6 (0,7)
OHL-Wissen/Kompetenz^h^	5,6 (1,1)
*Physische Mundgesundheit*	
DMF‑T	19,2 (5,3)
PhOX-Summenwert	82,3 (8,5)
PhOX Zahn	30,1 (6,7)
PhOX Weichgewebe	15,5 (1,5)
PhOX Kiefer	11,5 (0,8)
PhOX Funktion	13,9 (2,6)
PhOX Wahrnehmung	11,3 (2,0)

*DMF‑T* Decayed-Missing-Filled-Teeth-Index, *OHL* Oral Health Literacy, *PhOX* Physical Oral Health Index, *SF‑8* Short Form‑8 Health Survey

^a^ 224 fehlende Werte für Geschlecht

^b^ 338 fehlende Werte für Schulbildung

^c^ 566 fehlende Werte für Beschäftigungssituation

^d^ 628 fehlende Werte für Lebensqualität

^e^ 459 fehlende Werte für Parodontitistherapie

^f^ 284 fehlende Werte für professionelle Zahnreinigung

^g^ 322 fehlende Werte für Bonusheft

^h^ 413 fehlende Werte für OHL

### Mundgesundheitskompetenz und -verhalten im Zusammenhang mit physischer Mundgesundheit

Die PhOX-Summenwerte als Indikator für die physische Mundgesundheit lagen bei allen Teilnehmenden, die die 10 Fragen zu Mundgesundheitskompetenz und -verhalten (OHL) korrekt beantwortet hatten, höher als bei denjenigen, die die Fragen falsch beantwortet hatten oder nicht beantworten konnten (Tab. 2). Bis auf die Frage, ob eine Mundspülung nach der Anwendung heruntergeschluckt werden sollte (*p* = 0,034), waren alle Unterschiede sehr hoch signifikant. Die Effektgrößen reichten von 0,16 bezüglich des Wissens zum Zusammenhang zwischen Mund- und Allgemeingesundheit (Herzinfarkt) bis 0,50 bezüglich des Zahnarztbesuchs in den letzten 12 Monaten.

**Table 2:** 

Subskalen und Kriterien	Nicht korrekt/nicht gewusst	Korrekt	Effektgröße	*p*-Wert
	PhOX-Summenwert (SD)			
*Verhalten*				
Zeitpunkt letzter Zahnarztbesuch (innerhalb letzte 12 Monate)	78,7 (11,4)	82,8 (7,9)	0,50	< 0,001
Grund letzter Zahnarztbesuch (Vorsorge/Kontrolle)	80,5 (8,9)	83,4 (7,7)	0,36	< 0,001
Durchgeführte Häufigkeit Zähneputzen (mind. 2‑mal pro Tag)	81,2 (9,6)	82,7 (8,1)	0,18	< 0,001
*Kompetenz*				
Mundgesundheit und Erkrankungen (Herzinfarkt)	82,3 (8,4)	83,6 (7,8)	0,16	< 0,001
Kariesprotektiver Bestandteil Zahnpasta (Fluoride)	80,0 (9,8)	83,0 (7,9)	0,36	< 0,001
Empfohlene Häufigkeit Zähneputzen (2-mal pro Tag)	80,3 (9,5)	82,6 (8,3)	0,28	0,001
Kariogener Bestandteil Nahrung (Zucker)	77,8 (10,8)	82,7 (8,2)	0,59	< 0,001
Anwendungshinweis Mundspülung (nicht schlucken)	80,9 (7,9)	82,5 (8,4)	0,19	0,034
Anwendungshinweis Mundspülung (Wirkdauer)	81,1 (8,8)	83,4 (7,8)	0,28	< 0,001
Vorgehen bei Zahnfleischblutung (weiter putzen)	79,3 (9,7)	83,2 (7,8)	0,48	< 0,001

*PhOX* Physical Oral Health Index, *SD* Standardabweichung

Der Summenwert der 10 OHL-Fragen korreliert statistisch signifikant mit dem PhOX-Summenwert (r = 0,23; *p* < 0,001; Tab. 3). Die Größe des Effekts liegt entsprechend Cohen zwischen klein und moderat. Die Korrelationen der Subskalen *Verhalten* (r = 0,17) und *Kompetenz* (r = 0,19) lagen etwas darunter, waren aber dennoch statistisch signifikant (beide *p* < 0,001). Auch in den Subskalen des PhOX zeigten sich relevante Zusammenhänge (Tab. 3). Diese bestanden primär mit der Subskala *Zahngesundheit* und deutlich geringer noch mit der Subskala *Funktion*.

**Table 3:** 

OHL	PhOX	Zahn	Weichgewebe	Kiefer	Funktion	Wahrnehmung
	Gesamt					
*Pearson-Produkt-Moment-Korrelationen (p-Wert)*						
Gesamt	0,23 (< 0,001)	0,22 (< 0,001)	0,07 (< 0,001)	0,02 (0,159)	0,13 (< 0,001)	0,04 (0,010)
Verhalten	0,17 (< 0,001)	0,15 (< 0,001)	0,02 (0,116)	0,02 (0,117)	0,09 (< 0,001)	0,08 (< 0,001)
Kompetenz	0,19 (< 0,001)	0,18 (< 0,001)	0,07 (< 0,001)	0,01 (0,436)	0,10 (< 0,001)	0,00 (0,905)

*OHL* Oral Health Literacy, *PhOX* Physical Oral Health Index

Ein Anstieg von einem Punkt des OHL-Gesamtwerts war assoziiert mit einer Erhöhung des PhOX-Summenwerts um durchschnittlich 1,45 Punkte (Tab. 4). Dieser Zusammenhang reduzierte sich leicht, wenn für Alter und Geschlecht statistisch kontrolliert wurde auf 1,30 Punkte, wobei das Alter signifikant negativ mit dem PhOX-Summenwert assoziiert war. Das heißt, je höher das Alter war, desto schlechter war die Mundgesundheit. Der Zusammenhang zwischen OHL-Gesamtwert und PhOX-Summenwert änderte sich nur noch geringfügig, wenn zusätzlich Schulbildung und Beschäftigungssituation in das statistische Modell einbezogen wurden. Während kein Zusammenhang zwischen dem PhOX-Summenwert und der aktuellen Beschäftigungssituation bestand, so sank der PhOX-Summenwert mit jeder Stufe einer geringeren Schulbildung (Tab. 4).

**Table 4:** 

Modell	Prädiktor	PhOX gesamt	*p*-Wert
		Koeffizient (95 %-KI)	
#1	*OHL gesamt*	1,45 (1,28; 1,61)	< 0,001
#2	*OHL gesamt*	1,30 (1,14; 1,47)	< 0,001
	*Alter*	−0,22 (−0,25; −0,20)	< 0,001
	*Geschlecht*	0,22 (−0,22; 0,65)	0,330
#3	*OHL gesamt*	1,20 (1,02; 1,37)	< 0,001
	*Alter*	−0,21 (−0,24; −0,17)	< 0,001
	*Geschlecht*	0,40 (−0,07; 0,86)	0,095
	*Schulbildung*		
	Mind. 12 Jahre Schule^a^	–	–
	10 Jahre Schule	−1,71 (−2,23; −1,19)	< 0,001
	8 Jahre Schule	−3,16 (−3,77; −2,55)	< 0,001
	Ohne Abschluss	−5,69 (−7,19; −4,20)	< 0,001
	*Beruf*		
	Vollzeitbeschäftigt^a^	–	–
	Teilzeitbeschäftigt	−0,32 (−0,95; 0,31)	0,321
	Altersteilzeit oder Ruhestand	0,64 (−1,17; 2,44)	0,488
	Arbeitssuchend, in Ausbildung oder Fortbildung	0,38 (−0,28; 1,04)	0,257

*OHL* Oral Health Literacy, *PhOX* Physical Oral Health Index

^a^Referenzkategorie

## Diskussion

Die Ergebnisse dieser Studie legen nahe, dass ein Zusammenhang zwischen physischer Mundgesundheit und Mundgesundheitskompetenz und -verhalten besteht. Je höher die Werte der Teilnehmenden zur Mundgesundheitskompetenz und zum Mundgesundheitsverhalten ausfielen, desto besser war im Durchschnitt auch deren Mundgesundheit.

Die beobachteten Zusammenhänge erscheinen sehr plausibel. Mundgesundheit kann von vielen Aspekten beeinflusst werden, daher liegen kleine bis moderate Effekte vor. Die Ergebnisse für die einzelnen OHL-Aspekte befinden sich im vorher erwarteten Bereich. Die Studie stellt jedoch nur eine Momentaufnahme dar, da die aktuell erhobene physische Mundgesundheit aus der Akkumulation verschiedener Risiken resultiert, die in einer vorangegangenen Lebensphase mit anderen Einstellungen und Verhaltensweisen und anderen Lebensbedingungen aufgetreten sein können. Im Gegensatz dazu wird die OHL nur für den Moment bestimmt. Die Plausibilität des Zusammenhangs wird auch durch die Korrelationsmatrix bestätigt. Die höchsten und wesentlichen Korrelationen mit den OHL-Werten wurden für die Subskalen *Zahngesundheit* und *Funktion* gefunden. Das im OHL erfasste Wissen und Verhalten zielen vorrangig auf die Risiken für Karies, Parodontitis und damit letztendlich Zahnverlust ab. Die Prävalenz und Folgen dieser Erkrankungen sollten somit im Zusammenhang mit den OHL-Werten stehen, was auch durch die Ergebnisse bestätigt wird. Demgegenüber sind angeborene oder erworbene Veränderungen der Weichgewebe oder Kiefer nicht unmittelbar über Mundgesundheitskompetenz und -verhalten zu beeinflussen. Die geringen Zusammenhänge mit der Subskala *Wahrnehmung* können durch die geringe Prävalenz von Schmerzen und Missempfindungen erklärt werden, da es sich hier nicht um eine klinische Population handelt. Auch wenn sich aufgrund des Studiendesigns keine klare Aussage zur Richtung des Zusammenhangs treffen lässt, so erscheint es aus theoretischen Überlegungen wesentlich plausibler, dass Mundgesundheitskompetenz und -verhalten die Mundgesundheit beeinflussen und nicht vice versa. Auch wenn theoretisch denkbar akute orale Beschwerden eventuell die Frequenz des Zähneputzens oder die Inanspruchnahme von zahnärztlichen Leistungen beeinflussen könnten, dürfte dies – wenn überhaupt – nur einen sehr kleinen Teil der Studienpopulation betreffen. Außerdem wären damit nur einige Aspekte der OHL betroffen. Auch die Regressionsanalysen untermauern den gefundenen Zusammenhang. So sank der Regressionskoeffizient leicht, wenn potenzielle Confounder einbezogen wurden. Mit höherem Alter steigt die Prävalenz von kariesbedingten Folgen wie Füllungen oder Zahnersatz und damit sinkt auch die physische Mundgesundheit. Dies wurde im Regressionsmodell bestätigt. Auch der Bildungsgrad erklärte einen Teil der Varianz der PhOX-Werte. Trotz Einschluss dieser beiden wesentlichen Faktoren für die Mundgesundheit blieb der Effekt der OHL wesentlich. Betrachtet man die biologischen, pathophysiologischen und psychologischen Aspekte, erscheint eine weitgehend kausale Wirkung von Mundgesundheitskompetenz und -verhalten auf die physische Mundgesundheit sehr plausibel.

Frühere Studien bestätigen die Ergebnisse dieser Studie, dass Mundgesundheitskompetenz und -verhalten mit physischer Mundgesundheit assoziiert sind [6, 10, 23–25]. Konkret belegen die Studien Zusammenhänge der einzelnen Parameter der physischen Mundgesundheit wie Plaque-Index, DMFT-Index und Parodontitis mit Mundgesundheitskompetenz und -verhalten. In der vorliegenden Studie ist jedoch die physische Mundgesundheit mithilfe des PhOX erfasst, welcher verschiedene Parameter der physischen Mundgesundheit in einem einzigen Wert zusammenfasst. Somit konnten alle relevanten Parameter der physischen Mundgesundheit über den PhOX mit Mundgesundheitskompetenz und -verhalten in Korrelation gebracht werden, was eine höhere Aussagekraft zur Folge hat. Die DMS V zeigt zudem eine Abhängigkeit der Kariesverteilung vom sozialen Status. In dieser Studie konnte ein Zusammenhang zwischen physischer Mundgesundheit und Schulbildung gezeigt werden, was die Studie von Baskaradoss et al. [10] ebenfalls bestätigt.

Die Studie hat Stärken und Schwächen. Sie untersuchte den Zusammenhang zwischen der physischen Mundgesundheit und Mundgesundheitskompetenz und -verhalten in einer bevölkerungsbasierten umfangreichen Stichprobe von Männern und Frauen zwischen 45 und 74 Jahren. Die Stichprobe wurde randomisiert rekrutiert. Es wurden validierte Instrumente und Fragebögen verwendet, die in klinischen und epidemiologischen Studien verwendet und hinsichtlich ihrer Validität und Reliabilität bereits getestet wurden. Dadurch ist gewährleistet, dass die Ergebnisse dieser Studie mit anderen Studien vergleichbar sind. Das Untersuchungsprotokoll lief standardisiert ab und sämtliche Untersuchungen wurden von zertifiziertem und kalibriertem Studienpersonal durchgeführt. Die Analysen wurden für verschiedene Confounder adjustiert. Eventuelle Einschränkungen ergeben sich aus dem monozentrischen Studiendesign, das ausschließlich im Hamburger Einwohnermeldeamt registrierte Personen in die Studie einbeziehen lässt. Es ist jedoch davon auszugehen, dass durch die Größe Hamburgs als zweitgrößte Metropole Deutschlands und die hohe Fallzahl der Studie die Studienteilnehmer:innen auch für andere Großstädte Deutschlands repräsentativ sein dürften. Auch wenn nur 5510 Personen aus den 10.000 Teilnehmenden der HCHS-Baseline-Untersuchung an dieser Studie teilgenommen haben, sehen wir keinen Grund zu der Annahme, dass durch die Stichprobe der Zusammenhang zwischen Mundgesundheitsverhalten und -kompetenz und physischer Mundgesundheit wesentlich beeinflusst wurde.

Die Ergebnisse implizieren, dass im Sinne einer Verhaltensprävention eine Steigerung der Mundgesundheitskompetenz und des mundgesundheitsförderlichen Verhaltens zu einer besseren Mundgesundheit führt. Um dies in der regulären zahnmedizinischen Versorgung umzusetzen, können verschiedene Wege gegangen werden. Prinzipiell verstärken sich die Effekte von direkten Interventionen (z. B. Austeilen von Informationsmaterial bei den Patient:innen), wenn gleichzeitig auch die Ausbildung der zukünftigen Zahnärzt:innen verbessert wird. Vieles spricht dafür, dass Zahnärzt:innen eine zentrale Rolle bei der Verbesserung der Mundgesundheitskompetenz von Patient:innen einnehmen, da sie auch auf eine intrinsische Motivation der Patient:innen hinwirken können, Informationsmaterial auch zu lesen und zu berücksichtigen. Ausschließlich direkte Interventionen (z. B. nur Informationsmaterial) sind daher nicht nur zeitlich, sondern auch auf nur einen geringen Teil der Patient:innen beschränkt und damit weder effizient noch nachhaltig. Wenn im Zahnmedizinstudium ein verstärkter Fokus auf Mundgesundheitskompetenz und -verhalten gelegt wird, werden davon potenziell alle zukünftigen Patient:innen dieser Zahnärzt:innen profitieren. Ein wesentlicher Punkt dabei ist die Kommunikation der Inhalte. Studierende sollten daher nicht nur die theoretischen Grundlagen erlernen, sondern viel wichtiger noch die daraus resultierenden Anwendungen und Konsequenzen reflektieren, erproben und trainieren. Erste Ansätze wie im Modellstudiengang „Zahnmedizin iMED Dent“ am Universitätsklinikum Hamburg-Eppendorf sind sehr vielversprechend. Sicherlich muss angemerkt werden, dass primär die Personen mit Zugang zur zahnmedizinischen Versorgung profitieren werden. Damit auch Personen ohne diesen Zugang profitieren können, bedarf es weiterer Anstrengungen.

## Fazit

Zusammenfassend lässt sich sagen, dass die Verbesserung der Mundgesundheitskompetenz und des Mundgesundheitsverhaltens der Patient:innen dazu beitragen kann, die physische Mundgesundheit und die zahnärztlichen Behandlungsergebnisse insgesamt zu verbessern. Daher sollte die Verbesserung der Mundgesundheitskompetenz und des Mundgesundheitsverhaltens ein wesentlicher Bestandteil in der zahnmedizinischen Ausbildung sein.
